# 한국 중환자실 간호사 대상 고위험약물 투약오류 예방 임상의사결정 프로그램 효과: 비동등 대조군 전후설계 연구

**DOI:** 10.7586/jkbns.25.031

**Published:** 2025-08-22

**Authors:** Se Yeong Park, SoMi Park, Ga Young Kim, Seung Ah Hong, Hyang Ok Choi, Sunyoung Moon

**Affiliations:** 1 Department of Nursing Education Team, Wonju Severance Christian Hospital, Wonju, Korea; 1 원주세브란스기독병원 간호교육팀; 2 Wonju College of Nursing, Yonsei University, Wonju, Korea; 2 연세대학교 원주간호대학; 3 Division of Nursing, Wonju Severance Christian Hospital, Wonju, Korea; 3 원주세브란스기독병원 간호국

**Keywords:** Nurses, Intensive care units, Medication errors, Clinical decision-making, Simulation training, 간호사, 중환자실, 투약오류, 임상의사결정, 시뮬레이션교육

## Abstract

**Purpose:**

This study aimed to evaluate the effectiveness of a simulation based clinical decision-making program for preventing high-alert medication errors among intensive care unit nurses.

**Methods:**

A nonequivalent control group pre/post-test design was employed. The study involved 40 nurses working in the intensive care unit of a tertiary general hospital. The results for an experimental group (n = 20) that participated in the simulation-based clinical decision-making program for preventing high-alert medication errors (duration 180 minutes) were compared with those of a control group (n = 20) that received lecture education (duration 90 minutes). The intervention was conducted and data were collected from June 24 to October 31, 2024. Registered with the Clinical Research Information Service (KCT0010214).

**Results:**

The experimental group exhibited improved clinical decision-making ability (*χ*^2^ = 10.57, *p* = .005), reduced risk of high-alert medication errors (*χ*^2^ = 6.17, *p* = .046), and fewer experiences of near-miss medication errors (*χ*^2^ = 7.05, *p* = .008) compared to the control group immediately and 3 months after the intervention.

**Conclusion:**

The findings suggest that the program can be effectively used to improve the clinical decision-making ability and to reduce the risk of high-alert medication errors among intensive care unit nurses, thereby contributing to ensuring medication safety in intensive care units by reducing the occurrence of medication errors.

## 서론

### 1. 연구의 필요성

중환자실은 환자의 높은 중증도에 따른 다제약물 사용과 복잡한 투약 처방으로 투약오류가 가장 많이 발생한다[1-3]. 특히 중환자실에서 빈번하게 처방 및 사용되는 고위험약물 투약오류는 환자의 장기 손상뿐 아니라 사망까지 초래한다[3]. 그러나, 중환자실 고위험약물 처방의 92.5%에서 오류가 있었다[3]. 또한, 고위험약물을 포함한 적신호 사건은 병동보다 중환자실에서 발생 위험이 약 5배 더 높았다[4]. 그럼에도 불구하고 간호사가 투약과정 전반에서 올바른 지식을 활용한다면 투약오류 진행 차단을 통해 발생 가능한 투약오류의 최대 90%까지 예방할 수 있기에[5] 중환자실 간호사를 통한 고위험약물 투약오류 예방 전략이 마련되어야 한다.

투약오류 예측요인의 하나인 투약오류 위험수준은 약물에 대한 지식과 지식에 대한 확실성을 기반으로 오류 위험을 추정하는 것이다[6]. 올바른 지식과 높은 확실성을 가진 사람은 투약오류 위험이 낮지만, 잘못된 지식과 높은 확실성을 가진 사람은 투약오류 위험이 높다[7]. 그러나 중환자실 간호사는 고위험약물 사용이 빈번함에도 고위험약물 지식 수준이 낮고, 약물 용량 계산이나 약물 규정 검토 등 정확하지 않은 지식에 대한 확실성을 높이는 영역에서 저조한 수행을 보여, 투약오류 위험수준이 높다[8]. 따라서 중환자실 투약오류 예방을 위해서는 중환자실 간호사의 고위험약물 투약오류 위험수준을 낮추는 것이 중요하다.

의사결정은 불확실한 상황에서 목표 달성을 위해 사실에 근거한 판단을 하는 문제해결 과정으로, 의사결정자는 지식과 경험을 기반으로 문제해결에 필요한 정보와 자원을 수집, 분석하여야 한다[9]. 즉 간호사는 고위험약물 투약 시 불확실함을 느낄 경우, 문제해결을 위한 탐색을 시작하여 정확한 정보에 근거한 의사결정을 반복해야 한다. 이러한 간호사의 올바른 의사결정은 투약오류 위험수준 감소에 효과적일 것으로 보인다. 그러나 간호사의 투약오류 위험수준 선행연구는 조사연구가 대부분이고[6-8] 중환자실 고위험약물 투약 의사결정 교육 적용 연구는[10] 간호사의 투약안전역량 증진 효과를 보았으나 고위험약물 투약오류 위험수준 감소는 검증하지 않았다. 이에 본 연구는 고위험약물 투약오류 상황에서 간호사가 의사결정 탐색을 시작하고, 지식 확실성이 높아질 때까지 의사결정 순환 과정을 적용하는 교육을 개발하고 효과를 검증하고자 하였다. 또한, 고위험약물 지식과 약물계산 자신감은 간호사 경력에 따른 차이가 없고 경력이 증가함에 따라 저절로 획득되지 않기에[11] 중환자실 고위험약물 투약 의사결정 교육은 전체 경력간호사를 대상으로 적용되어야 한다. 그러나 선행연구[10]에서는 교육 대상을 저연차 간호사로 국한하였기에, 본 연구에서 전체 중환자실 경력간호사를 대상으로 교육 개발 및 적용을 시도하였다.

시뮬레이션 교육은 임상의 오류 환경을 연출하여 간호사에게 경험학습과 디브리핑 성찰학습을 제공함으로써 학습 경험을 실무에서 전환하도록 하는 전략이다[12]. 시뮬레이션 교육은 실제 대상자에게 위험을 가하지 않으면서 간호사가 안전한 환경에서 간호 수행을 반복 연습할 수 있도록 하기에[12] 오류가 발생하면 안 되는 수혈[13], 투약[14] 분야에서 유용한 교육방법으로 활용된다. 고위험약물 투약오류는 발생 시 적신호 사건으로 취급되기에 임상에서 간호사의 연습과 훈련이 제한되므로[14], 시뮬레이션을 활용한 교육 전략을 마련할 수 있다. 그러나 선행연구에서는[10] 간호사 의사결정 훈련의 효과적인 전략으로 보고된 사례기반학습과 시뮬레이션을 함께 적용하여 의사결정능력 향상 효과를 검증하였으나, 단일 중재로서 시뮬레이션 교육 효과를 검증한 연구는 거의 없다. 이에 본 연구에서는 중환자실 간호사를 위하여 시나리오 난이도와 응급성을 점진적으로 높여가는 연속된 시나리오 기반의 임상의사결정 프로그램을 개발하고 효과를 검증하고자 하였다.

시뮬레이션 상황은 오류를 발견, 차단하여 교정하는 과정을 통해 환자에게 위협이 될 수 있는 부정적 결과를 전환시킬 수 있다[15]. 선행연구에 의하면 간호대학생 대상 시뮬레이션 교육을 통하여 투약[11]과 수혈[13]에서의 오류 복구수준을 조사하여 이들의 환자안전 수행 수준이 높지 않음을 파악하였고, 이를 통해 향후 교육 방향을 제고해보고자 하였다. 간호사를 대상으로 고위험약물 투약오류 복구수준을 확인한 Ko와 Kim[14]의 연구에서는 신규와 상급초보 임상경력 단계 간호사만을 대상으로 이들의 고위험약물 투약오류 복구수준이 높지 않음을 확인하였다. 그러나 전체 중환자실 경력간호사에게 고위험약물 투약오류 복구 경험을 제공할 수 있는 시뮬레이션 교육을 적용하고 이들의 시뮬레이션 수행에서 고위험약물 투약오류 복구수준을 조사한 경우는 거의 없으므로, 본 연구를 통해 중환자실 고위험약물 투약안전 현황을 파악하여 지속적인 중환자실 고위험약물 투약안전 교육 전략 마련의 기초자료를 확보하고자 하였다.

이상의 연구 필요성에 따라 본 연구는 고위험약물 투약오류 예방 임상의사결정 프로그램이 중환자실 간호사의 임상의사결정능력, 고위험약물 투약오류 위험수준, 투약근접오류 경험에 미치는 효과를 확인하고 고위험약물 투약오류 복구수준을 조사하고자 하였다.

### 2. 연구의 목적

본 연구는 고위험약물 투약오류 예방 임상의사결정 프로그램을 개발하고 효과를 평가하기 위함이며, 연구가설은 다음과 같다.

1) 제1가설: 고위험약물 투약오류 예방 임상의사결정 프로그램에 참여한 실험군(이하 실험군)은 고위험약물 투약안전 이론교육에 참여한 대조군(이하 대조군)보다 시간 경과에 따라 임상의사결정능력이 높을 것이다.

2) 제2가설: 실험군은 대조군보다 시간 경과에 따라 고위험약물 투약오류 위험수준이 낮을 것이다.

3) 제3가설: 실험군은 대조군보다 투약근접오류 경험이 낮을 것이다.

## 연구 방법

### 1. 연구 설계

본 연구는 고위험약물 투약오류 예방 임상의사결정 프로그램이 중환자실 간호사의 임상의사결정능력, 고위험약물 투약오류 위험수준, 투약근접오류 경험에 미치는 효과를 검증하기 위한 비동등 대조군 전후설계의 유사실험 연구이다.

### 2. 연구 대상

본 연구는 다음 조건을 충족하는 이를 연구대상으로 하였다. (1) 본 연구목적을 이해하고 자발적 참여를 동의한 자, (2) 상급종합병원 성인중환자실 간호사, (3) 임상경력 12개월 이상인 자로, 이는 임상경력 12개월 미만 신규간호사는 투약지식이 부족하고, 환자안전 교육 기회가 부족함을 고려한 것이다[16].

본 연구 표본 수는 G*power 3.1.9.7 프로그램을 이용하여 두 집단 간 시간 경과에 따른 중재 효과를 평가하기 위한 반복측정 분산분석을 이용, 유의수준 .05, 검정력 .80, 효과크기는 0.50로 산출된 전체 표본 수는 36명이었다. 효과크기 산출근거는 간호대학생의 투약안전 시뮬레이션 교육[17]을 근거로 추정하였고, 10% 탈락율을 적용하여 총 40명 연구 대상자를 최종 산출하였다. 실험군과 대조군 중 탈락자는 없었으며, 최종 분석에 포함된 대상자는 실험군 20명, 대조군 20명으로 총 40명이었다.

### 3. 연구 도구

#### 1) 일반적 특성

일반적 특성은 나이, 성별, 근무부서, 임상경력, 현 부서경력, 최종학력을 조사하였다.

#### 2) 임상의사결정능력

임상의사결정능력은 Jenkins [18]가 간호사의 임상의사결정능력 측정을 위해 개발한 도구를 Baek [19]이 수정, 번역한 도구를 사용하여 대상자가 자가 보고한 점수로 측정하였다. 도구는 대안과 선택조사, 가치와 목표에 대한 검토, 결론에 대한 평가와 재평가, 정보에 대한 조사와 새로운 정보에 대한 일치화의 4개 하위 항목, 총 40개 문항으로 각 문항 점수는 ‘전혀 그렇지 않다’ 1점에서 ‘매우 그렇다’ 5점의 5점 Likert 척도로 점수가 높을수록 임상의사결정능력이 높음을 의미한다. 개발 당시[18] Cronbach’s α는 .83, Baek [19]의 연구 Cronbach’s α는 .77이었으며, 본 연구 Cronbach’s α는 .87이었다.

#### 3) 고위험약물 투약오류 위험수준

고위험약물 투약오류 위험수준은 Hsaio 등[20]이 개발한 고위험약물 지식 도구를 Kim과 Jung [8]이 수정•보완한 도구를 사용하여 대상자가 자가 보고한 점수를, Simonsen 등[7]이 개발한 투약오류 위험 산출 방법을 사용하여 측정하였다. 고위험약물 투약오류 위험수준은 관련 지식의 정확성에 대한 확신 정도를 통해 산출하며 지식 측정과 동일한 질문지를 이용하여 각 문항별로 ‘매우 불확실하다’ 1점, ‘불확실하다’ 2점, ‘확실하다’ 3점, ‘매우 확실하다’ 4점으로 측정하고, 지식 정확성 확신 정도에서 정답 여부를 뺀 차이 값으로 계산하며 점수가 높을수록 투약오류 위험수준이 높음을 의미한다. Kim과 Jung [8] 연구 Cronbach’s α는 .92, 본 연구 Cronbach’s α는 .88이었다.

#### 4) 투약근접오류 경험

투약근접오류 경험은 Park과 Lee [16]의 투약근접오류 경험 문항을 사용하여 대상자가 자가 보고한 점수로 측정하였다. 간호사가 경험한 투약에서의 근접오류 항목마다 ‘1회’당 1점, ‘5회’ 이상은 5점으로 측정한 것으로 점수가 높을수록 투약 근접오류 경험이 많은 것을 의미한다.

#### 5) 고위험약물 투약오류 복구 수준

고위험약물 투약오류 복구 수준은 2명의 훈련된 평가자가 본 연구를 위해 개발된 ‘중환자실 고위험약물 투약오류 복구 수행 관찰 체크리스트’를 활용하여 관찰 평가한 평균 점수로 측정하였다. 점수는 체크리스트 각 항목을 시행한 경우 2점, 시행하지 않은 경우 0점, 항목에 따라 부분 점수 1점을 주었고, 시나리오별 체크리스트 점수 합산 후 100점으로 환산하여 2명의 평가자 점수를 평균한 값으로 산출하였다. 점수가 높을수록 고위험약물 투약오류 복구가 잘 수행됨을 의미한다.

본 연구를 위해 개발된 체크리스트에 대한 신뢰도는 서로 다른 2인이 동일한 관찰에 비슷한 점수를 주는 정도를 확인하는 평가자간 신뢰도로 확인하였다. 이를 위해 급내 상관계수(intraclass correlation coefficients)를 이용하였으며 분석결과 개발된 시나리오 1, 2, 3, 4에서 각각 .97, .85, .96, .90으로 나타나 급내 상관계수가 .80일 때 평가자간 평가가 일치되는 것으로 판단할 수 있다는 기준을 충족하였다[21]. 이에 중환자실 고위험약물 투약오류 복구 수행 관찰 체크리스트는 모두 평가자간 신뢰도가 적절한 것으로 검증되었다(Table 1).

### 4. 고위험약물 투약오류 예방 임상의사결정 프로그램 개발 및 구성

본 연구의 중재 개발 및 적용은 ADDIE (Analyze, Design, Development, Implement, Evaluate) 모형[22]을 기반으로 하였다(Table 2).

#### 1) 분석(Analyze)

분석 단계는 교육 필요성을 확인하고 요구분석을 하는 단계[22]로 본 연구에서는 2015년 이후 문헌을 고찰하였다. 국내∙외 논문을 검색어로 12편 검색하였고, 추가 수기검색으로 3편 문헌을 추가하여 총 15편 문헌을 분석하였다. 문헌고찰을 통해 중환자실 고위험약물 투약오류 예방 교육 개발 필요성을 확인하였으며[3,4,14,23], 중환자실 경력간호사를 교육 대상으로 하였다[8,11].

#### 2) 설계(Design)

설계 단계는 교육목표를 명세화하고 평가도구를 설계하며 교수 전략을 선정하는 단계[22]이다. 본 연구에서는 간호사의 고위험약물 투약오류 위험 수준이 투약오류의 주요 예측요인임을 고려하여[6] 간호사의 임상의사결정 훈련을 계획하면서 교육목표를 고위험약물 투약오류 위험 수준 저하와 임상의사결정능력 향상, 투약근접오류 경험 감소로 수립하였고 이에 따라 평가도구를 선정하였다. 선행문헌 고찰결과에 따라 중재 전략과 강도를 구성하였는데, 고충실도 시뮬레이터를 활용한 시뮬레이션은 실제와 유사하면서 오류가 발생해도 안전한 상황을 연출할 수 있기에, 학습자의 단순한 술기 기반 오류 뿐 아니라 임상 상황에서 올바르지 못한 판단이나 정확하지 않은 지식으로 인한 오류를 확인할 수 있는 방법으로[13] 간호사의 투약안전역량과 임상의사결정능력 증진에 효과가 있었다[10]. 이에 본 연구에서는 고충실도 시뮬레이터를 활용한 시뮬레이션 교육을 중재 전략으로 선정하였다. 간호사의 환자안전 시뮬레이션 교육 선행연구[14,24]에서는 평균 시뮬레이션 구동 횟수 약 3회, 회당 디브리핑 포함 시뮬레이션 구동 시간 약 60분, 중재 기간 1∼2일, 중재 횟수 1회로 제공되었기에 본 연구는 시뮬레이션 구동 횟수 3회, 디브리핑 포함 회당 시뮬레이션 구동 시간은 약 60분의 총 1일 1회의 180분 교육을 중재 강도로 구성하였다.

#### 3) 개발(Development)

개발 단계는 교육자료를 개발하고, 사전 형성평가를 통해 교육을 수정, 보완하는 단계[22]이다. 본 연구에서는 간호사의 임상판단 능력이 부족하면 오류가 복구되지 않는 상황으로 시나리오를 작성하여, 간호사가 오류확인-중단-교정단계를 적용하도록 시나리오를 구성하고[13-15], 시나리오 난이도는 단순에서 복잡으로, 상황 전개는 일반적인 상황에서 응급 상황으로 진행되도록 하였다[25]. Park의 연구[10]에서는 중환자실 고위험약물 투약 상황에서의 올바른 임상의사결정 탐색-설계-선택을 반영하는 항목을 개발하였고, 이를 기반으로 중재를 적용하여 중환자실 상급초보 간호사의 임상의사결정능력 증진 효과를 검증하였기에, 연구자는 문헌고찰 결과와 Park의 임상의사결정 구성요소 항목[10]을 기반으로 시뮬레이션 시나리오에서 학습자가 올바른 의사결정을 하지 않는다면 오류가 복구되지 않는 중환자실 고위험약물 투약오류 상황 20문항을 도출하였다.

기초 설계된 중환자실 고위험약물 투약오류 문항에 대한 전문가 타당도 검증을 위해 간호학과 교수 2인, 상급종합병원 간호교육팀 간호관리자 2인, 중환자실 간호관리자 3인, 총 7인의 전문가 집단에게 2024년 6월 5일부터 2024년 6월 18일까지 전문가 내용타당도 검증을 받았다. 총 20개의 문항은 4점 Likert 척도로 평가하여 내용타당도 지수(Content Validity Index, CVI)를 산출하였고, 전문가에게 문항별 의견 및 보완사항을 추가로 기입하도록 하였다. 전문가 집단이 6명에서 10명인 경우는 내용타당도 지수가 0.78 이상이면 문항의 내용타당도가 만족된 것으로 보기에[26] CVI 값이 0.86인 문항 5개와 1.0인 문항 15개의 총 20개 문항을 중환자실 간호사의 고위험약물 투약오류 복구 수행 문항으로 포함하였다. 이후 전문가 의견을 반영하여 수정, 보완 후 최종 고위험약물 투약오류 복구 수행 체크리스트를 확정하고, 개발된 체크리스트를 기반으로 시뮬레이션 시나리오 내용, 시나리오 내 진행 순서, 시나리오별 경험의 내용을 구성하였다. 시뮬레이션 시나리오는 고농도전해질 투약오류 유발 상황, 인슐린 투약오류 유발 상황, 고위험약물이 투여되는 두 명의 환자가 동시에 간호요구를 하며 투약오류가 유발되는 상황으로 점차 복잡하고 긴급해지는 시나리오 흐름으로 구성하였다.

프로그램 개발 후 전문가 내용 타당도와 예비교육을 통한 학습자 타당도를 조사하였다. 전문가 타당도는 간호학과 교수 1인, 상급종합병원 간호교육관리자 2인, 석사학위 이상 교육간호사 2인에게 검증받았다. 전문가 타당도 결과, 고농도전해질과 인슐린 외 중등도 진정의약품과 신경근 차단체 고위험약물을 시나리오에 추가하자는 의견과 교육 참여자가 경력간호사이므로 네 번째 시나리오에서는 난이도를 더 복잡하고 응급한 상황으로 연출하는 것이 필요하다는 의견이 있었다. 이에 최종 시뮬레이션 시나리오를 고농도전해질 투약오류 유발 상황, 인슐린 투약오류 유발 상황, 고위험약물이 투여되는 두 명의 환자가 동시에 간호 요구를 하며 투약오류가 유발되는 상황, 중환자실 응급상황 발생으로 고위험약물인 중등도 진정의약품과 신경 차단제가 투여되며 투약오류가 유발되는 상황의 총 4개 시나리오를 개발하였으며, 시나리오 흐름이 전개됨에 따라 난이도가 복잡하고 긴급해지도록 구성하여 최종 수정, 완성하였다. 수정, 보완하여 완성된 최종 중재로 첫 번째 학습자 타당도 조사를 실시하여 사전브리핑과 시뮬레이션 구동, 디브리핑 운영 시간을 조정하였고 이후 추가 예비조사를 통해 교육 운영에 특별한 문제가 없음을 확인하여 중재를 최종 확정하였으며, 중재는 총 사전브리핑 30분, 시뮬레이션 시나리오 구동 50분, 디브리핑 100분으로 구성된 총 180분의 시뮬레이션 프로그램이다(Table 3).

#### 4) 실행(Implement)

실행 단계는 개발된 프로그램을 적용하는 단계[22]이다. 본 연구에서 자료 수집 및 중재 적용은 원주세브란스기독병원 교육공간과 통합시뮬레이션센터에서 이루어졌으며, 중재 제공의 일관성을 위해 훈련된 연구 중재자가 중재를 제공하였다. 연구 중재자의 중재 적용에서의 편차를 줄이기 위하여 본 연구에서는 중재 프로토콜을 개발하고 중재자 훈련을 하였다. 또한, 중재 적용 시 고위험약물 투약오류 복구수준 평가의 중재자 간 일관성을 높이기 위하여 개발된 체크리스트를 활용한 시뮬레이션 구동 및 디브리핑 성찰학습 운영 훈련을 진행하였고, 이를 통해 평가자 간 신뢰도를 확보하였다. 연구자는 자료수집에는 직접 참여하지 않고, 연구 진행절차 및 자료수집 절차를 프로토콜로 만들고 자료수집자를 훈련하여 진행, 중재 효과 측정의 타당성을 확보하고자 하였다. 실험군과 대조군 집단 간 확산 효과 예방을 위하여 연구 종료까지 연구 대상자에게 자신의 배정 집단에 대한 정보를 공개하지 않았고, 중재 시 제공되는 교육자료와 교육내용을 외부에 노출하지 않도록 하였으며 대조군 교육과 실험군 중재는 시차를 두고 적용하고, 실험군과 대조군이 중재 및 교육기간 동안 마주치지 않도록 교육 적용일과 교육 동선, 교육공간을 구분하였다.

실험군 중재는 각 시나리오의 사례 개요 및 실습 환경에 대한 사전브리핑 제공 후 시나리오 구동과 디브리핑 진행으로 적용되었다. 실험군은 첫 번째와 두 번째 시나리오에서 한 명의 환자를 대상으로 고농도전해질과 인슐린 투약오류를 복구하는 시나리오 구동을 각 10분, 이후 디브리핑 성찰학습을 각 20분씩 진행하였다. 이후 세 번째와 네 번째 시나리오에서는 두 명의 환자를 동시에 간호하는 상황에서 고농도전해질과 인슐린 투약오류, 신경근 차단제 투약오류를 포함한 저혈압과 혈역학적 불안정 상황을 제시하여 시나리오의 응급성과 난이도를 높였다. 각 시나리오는 15분씩 실습하고, 디브리핑 성찰학습을 각 30분씩 진행하였다(Table 2). 실험군의 시나리오 구동은 고위험약물 투약오류 복구를 위해 임상의사결정 단계를 적용하는 경험학습을 제공하였고, 디브리핑은 이들이 수행한 임상의사결정 과정에 대한 평가와 더 나은 임상의사결정 적용에 대한 피드백 과정을 위한 성찰을 촉진하기 위해 적용되었다(Table 3).

대조군 교육은 본 연구를 위해 개발된 이론교육으로 중환자실 고위험약물 투약안전과 투약 상황에서의 임상의사결정, 올바른 임상의사결정을 적용한 고위험약물 투약오류 복구, 고위험약물 투약지식에 대한 불확실성이 있는 경우 탐색과 설계에 활용할 수 있는 자원과 정보에 대한 것을 내용으로 포함하였다. 중재자는 대조군 4∼6명을 대상으로 1회 90분 이론교육을 적용하였으며, 교육 전과 후로 교육자료를 제공하였고, 대조군 교육 후 실험군을 2024년 7월 30일, 31일 이틀 중 1회 180분의 고위험약물 투약오류 예방 임상의사결정 프로그램에 각자 한 번씩 참석하도록 하였다.

#### 5) 평가(Evaluation)

평가 단계는 교육 성과를 평가하는 단계[22]로 본 연구의 대상자는 연구 참여 동의 후 실험군 또는 대조군에 배정되었고, 사전조사와 교육 중재, 사후조사에 참여하였다. 2024년 6월 24일부터 6월 30일까지 사전조사가 진행되고 사전조사 후 대조군은 고위험약물 투약안전 이론교육을 받고, 교육 직후 사후조사 1(사전조사 2주 후)에 참여하였다. 중재자는 실험군에게 중재를 적용하고, 중재 직후 사후조사 1(사전조사 2주 후)을 실시하였다. 실험군과 대조군 모두 사후조사 1의 3개월 후 사후조사 2를 실시하였다. 사전조사와 사후조사 1은 같은 도구의 반복측정 최소 간격 2주를 유지하였고[27], 교육의 지속효과 확인 시기는 간호사 투약근접오류 경험에 대한 선행연구를[16] 근거로 하였다.

### 5. 자료 수집

개발된 프로그램 적용 및 평가를 위한 자료 수집 절차는 ADDIE 모형[22]의 실행(Implement)과 평가(Evaluation) 단계를 기반으로 진행되었다(Table 2).

### 6. 자료 분석

자료분석은 SPSS version 23.0 (IBM Corp, Armonk, NY, USA)을 이용하였다. 대상자의 일반적 특성은 기술통계 방법을 이용하여 실수와 백분율, 평균과 표준편차를 산출하였고, 정규성 검정은 Shapiro-wilk test를 이용하였다. 실험군과 대조군의 사전 동질성 검정은 명목변수의 경우 *χ*^2^-test, Fisher’s exact test를, 연속변수의 경우 정규분포 여부에 따라 independent t-test 또는 Mann-whitney U test로 분석하였다. 중재 효과에 대한 가설검정으로 실험군과 대조군의 시간 경과에 따른 변수 차이는 generalized estimating equations (GEE)로 분석하였는데, GEE 분석은 두 집단에서 시간에 따른 종속변수의 차이를 비교할 수 있으며 정규성 가정에서 자유롭다는 장점이 있다[28]. 본 연구의 통계적 유의수준은 *p* < .05에서 검정하였다.

### 7. 윤리적 고려

본 연구는 연세대학교 원주세브란스병원 연구윤리심의위원회의 승인(CR324008)을 받아 시행되었고, 질병관리청이 운영하는 임상연구정보서비스(Clinical Research Information Service)에 등록되었다(KCT0010214). 연구자는 자료수집 전 대상자에게 연구배경과 목적을 설명하고, 참여자의 자발적 동의와 서면 동의서 작성 후 연구를 진행하였다. 연구 설명문에는 윤리적 고려와 관련된 내용을 기술하고, 수집된 모든 정보는 익명으로 처리되고 기밀유지가 되며, 연구 참여로 인해 발생하는 이익과 위험, 연구 철회가 가능함, 이로 인한 불이익이 없음을 설명하였다. 연구 참여 여부나 작성한 설문지의 내용은 관리자나 간호국에 노출되지 않도록 연구자가 직접 밀봉하여 배부, 수거하였고 대상자를 식별할 수 있는 불필요한 개인정보는 수집하지 않았으며 개인정보를 식별할 수 있는 정보 중 수집해야 하는 정보(나이, 근무부서, 임상경력, 성별 등)에 대해선 이중으로 코드화하여 기밀을 유지하였고 연구 참여자에게 사전 설명하였다. 수집된 자료는 연구목적 이외에 사용되지 않고 연구자만 알 수 있는 컴퓨터에 보완 암호를 책정하여 보관되고 코드화하여 다뤄질 것과 연구결과가 작성, 발표되는 경우 개인정보가 공표되지 않는 것, 연구 완료 시점까지 수집 자료를 보관하고 3년간 보관 후 종이 자료는 분쇄기로 파기, 전자 자료는 영구 삭제할 것을 사전 연구 참여자에게 설명하였고 이행할 것이다. 연구 종료 후 대조군 중 희망자에 한해 개발된 프로그램이 추가 제공될 수 있음을 설명하였다.

## 연구 결과

### 1. 대상자의 일반적 특성 및 실험군과 대조군의 동질성 검정

연구대상자의 일반적 특성과 종속변수인 임상의사결정능력, 고위험약물 투약오류 위험수준, 투약근접오류 경험 횟수에서 모두 실험군과 대조군 사이에 통계적으로 유의한 차이가 없어 두 집단 간 사전 동질성이 확보되었다(Table 4).

### 2. 고위험약물 투약오류 예방 임상의사결정 프로그램 효과 검증

1) 제1가설: ‘실험군은 대조군보다 시간 경과에 따라 임상의사결정능력이 높을 것이다’는 실험군과 대조군 간 임상의사결정능력의 점수가 중재 전(사전조사) 실험군 139.90 (± 7.41)점, 대조군 136.80 (± 9.45)점, 중재 직후 실험군 151.80 (± 13.77)점, 대조군 139.50 (± 8.70)점, 중재 3개월 후 실험군 145.20 (± 8.60)점, 대조군 138.45 (± 7.48)점으로 그룹(*χ*^2^ = 9.30, *p* = .002)과 시점(*χ*^2^ = 27.37, *p* < .001)에서 통계적으로 유의한 차이가 있었고, 시점에 따른 임상의사결정능력에서 집단 간 유의미한 교호작용이 확인되어(*χ*^2^ = 10.57, *p* = .005) 지지되었다.

2) 제2가설: ‘실험군은 대조군보다 시간 경과에 따라 고위험약물 투약오류 위험수준이 낮을 것이다’는 실험군과 대조군 간 고위험약물 투약오류 위험수준의 점수가 중재 전(사전조사) 실험군 1.58 (± 0.22)점, 대조군 1.59 (± 0.19)점, 중재 직후 실험군 1.39 (± 0.17)점, 대조군 1.51 (± 0.21)점, 중재 3개월 후 실험군 1.31 (± 0.16)점, 대조군 1.48 (± 0.26)점으로 그룹(*χ*^2^ = 4.08, *p* = .043)과 시점(*χ*^2^ = 33.72, *p* < .001)에서 통계적으로 유의한 차이가 있었고, 시점에 따른 고위험약물 투약오류 위험수준에서 집단 간 유의미한 교호작용이 확인되어(*χ*^2^ = 6.17, *p* = .046) 지지되었다.

3) 제3가설: ‘실험군은 대조군보다 투약근접오류 경험이 낮을 것이다’는 실험군과 대조군 간 투약근접오류 경험의 점수가 중재 전(사전조사) 실험군 9.80 (± 13.79)점, 대조군 3.50 (± 5.69)점과 중재 3개월 후 실험군 2.45 (± 3.07)점, 대조군 5.00 (± 4.55)점으로 그룹 간(*χ*^2^ = 1.09, *p* = .297), 시점(*χ*^2^ = 3.08, *p* = .079)에서 통계적으로 유의한 차이는 없었지만, 시점에 따른 투약근접오류 경험에서 집단 간 유의미한 교호작용이 확인되어(*χ*^2^ = 7.05, *p* = .008) 지지되었다(Table 5).

### 3. 중환자실 간호사의 고위험약물 투약오류 복구수준

중환자실 고위험약물 투약오류 복구수준은 총 4명으로 구성된 5개 조가 각각 난이도와 복잡함이 고조되는 연속된 시나리오를 경험하는 가운데 측정되었다. 연구 참여자는 돌아가며 담당간호사와 동료간호사 역할을 하였고, 시나리오 난이도가 심화되고 응급상황이 발생한 경우 팀원들이 함께 의논하여 의사결정을 통한 고위험약물 투약오류 복구 수행을 하였다. 전체 5개 조가 4개 시나리오를 경험하는 동안 고위험약물 투약오류 복구를 모두 수행하여 투약오류 발생없이 투약 수행을 마친 조는 하나도 없었으며, 모든 시나리오에서 투약오류가 발생하였다.

시나리오 1의 전체 고위험약물 투약오류 복구수준 점수는 100점 만점에 평균 45.00 (± 0.90)점, 최하 점수 35.42점, 최고 점수 56.25점이었다. 시나리오 2의 전체 고위험약물 투약오류 복구수준 점수는 평균 55.91 (± 0.94)점, 최하 점수 45.45점, 최고 점수 63.64점이었다. 시나리오 3의 전체 고위험약물 투약오류 복구수준 점수는 평균 56.50 (± 0.80)점, 최하 점수 52.50점, 최고 점수 62.50점이었다. 시나리오 4의 전체 고위험약물 투약오류 복구수준 점수는 평균 50.28 (± 0.82)점, 최하 점수 34.72점, 최고 점수 65.28점이었다(Table 1).

## 논의

본 연구는 중환자실 간호사에게 고위험약물 투약오류 예방 임상의사결정 프로그램을 적용하여 임사의사결정능력, 고위험약물 투약오류 위험수준, 투약근접오류 경험에 미치는 효과를 확인하기 위한 목적으로 시도되었다.

연구결과 실험군의 임상의사결정능력은 대조군보다 시간 경과에 따라 향상되었다. 이는 중환자실 상급초보 간호사에게 투약 상황에서의 의사결정 탐색-설계-선택 과정의 의사결정 훈련을 제공하여 의사결정능력 향상 효과를 본 선행연구와 유사한 결과이다[10].

본 연구 중재는 중환자실 간호사가 고위험약물 투약 상황에서 오류가 유발될 수 있는 위험을 인지하고 투약과정의 진행을 중단 및 교정하는 과정에서 의사결정 탐색-설계-선택 과정을 적용하도록 하였다. 또한, 시뮬레이션을 통하여 학습자가 수행한 의사결정 결과에 대한 평가와 피드백 순환 과정을 반복 경험하도록 하였다. 즉, 중환자실 간호사에게 임상의사결정 과정에 대한 반복 경험학습을 제공한 점이 임상의사결정능력 향상 효과로 이어졌다. 이는 의사결정 모델이 간호사의 의사결정능력 증진에 효과적인 이론적 기틀임을 입증한 선행연구[10]와 맥락을 같이 하는 결과로서, 임상간호사의 중재 이론으로 의사결정 모델의 유용성을 확인했다는데 의미가 있다.

간호사의 의사결정은 간호 상황에서 발생하는 문제의 원인을 규명하고 해결하는 과정으로 인지와 행동 훈련으로 강화할 수 있다[10]. 사례기반학습과 시뮬레이션 교육은 간호사 의사결정 교육의 유용한 전략으로 알려져 있기에[12] 선행연구[10]에서는 사례기반학습을 통한 해석강화 인지 훈련을 선행학습으로 제공한 후, 시뮬레이션 행동 훈련 적용으로 간호사의 임상의사결정능력 향상을 확인하였다. 그러나 본 연구에서는 시뮬레이션 교육만을 중재로 하여 고위험약물 투약상황에서의 의사결정에 대한 직접 수행을 통한 경험학습과 디브리핑 성찰학습으로 인지, 행동학습을 가능하게 하였다. 또한, 점차 난이도를 높인 연속된 시나리오 경험을 통해 반복된 의사결정 훈련을 제공하였기에 간호사의 의사결정능력 향상에 효과를 보았다. 이는 간호사의 의사결정능력 향상에 시뮬레이션 교육이 단일 중재로도 효과적임을 규명한 것이다. 이를 통해 임상에서 재현이나 반복 연습이 제한되면서도, 실수가 허용되지 않는 고위험약물 투약 교육과 같은 영역에서 시뮬레이션 교육의 활용 가능성을 확인하였다. 즉, 안전한 환경에서의 진행되는 간호사의 시뮬레이션 반복 교육은 실무에서 고위험약물 투약오류 발생 시 간호사의 올바른 의사결정을 이끌어 안전한 투약 수행을 하는데 도움이 될 것이다.

본 연구결과 실험군의 고위험약물 투약오류 위험수준은 대조군보다 시간 경과에 따라 낮아졌다. 선행연구에서 중환자실 간호사는 고위험약물 투약지식이 부족하고, 투약오류 위험수준이 높았다[8]. 이에 본 연구는 문헌고찰 결과에 따라 간호사의 고위험약물 투약오류 예방을 위한 교육 개발에 있어 중재 대상을 중환자실 간호사로 선별하고, 고위험약물 투약오류 위험수준을 낮추는데 중점을 둔 중재 개발을 시도하였다. 간호사의 고위험약물 투약오류 위험수준을 낮추기 위해서는 이들 스스로 고위험약물 투약지식의 확실성을 높이는 방법을 강구하도록 하여야 한다[6-8]. 이에 본 연구에서는 실제 고위험약물 투약오류 사례를 활용하여 중환자실 간호사가 고위험약물 투약오류 위험을 인지하도록 경각심을 일깨우고, 고위험약물 투약상황에서 불확실함을 느끼거나 자신의 지식과 경험에 확신이 없는 경우 관련 지침과 규정, 약물 정보, 주변 동료 등을 자원으로 활용하여 의사결정 탐색을 시작하도록 교육하였다. 또한, 의사결정 과정 적용 후에도 교육생이 예상한 결과가 나타나지 않는 경우 피드백 과정을 통해 다시 의사결정 탐색을 시작하고 의사결정을 반복하도록 시뮬레이션 시나리오를 구성하였다. 즉, 본 연구에서 간호사에게 의사결정에서 필요한 자원과 정보를 모색하는 탐색 과정[9]에 대한 훈련을 강화하고, 자신의 탐색 과정에 대한 성찰이 이뤄지도록 시나리오 반복 경험을 제공한 점이 투약오류 위험수준 감소 효과로 나타났다.

본 연구의 실험군은 대조군보다 중재 3개월 후 투약근접오류 경험이 감소하였다. 간호사 대상 교육으로 투약근접오류 감소 효과를 검증한 연구는 거의 없기에 직접적인 비교는 어려우나, 이는 중환자실 상급초보 간호사에게 의사결정 훈련을 적용하여 투약오류 예방에 활용되는 간호사의 능력인 투약안전역량 향상 효과를 검증한 선행연구 결과와 유사한 결과이다[10]. 투약근접오류는 투약과정에서의 오류로 인해 환자에게 해가 될 수 있었으나 상해나 위해가 생기지 않은 것[16]으로 위해 사건, 사망과 같은 중대 사고로 이어지기에[29] 예방이 중요하다. 그러나 선행연구에서는 환자안전 중재를 통해 간호사의 투약지식[14], 문제해결능력[24], 자신감[25] 향상은 확인하였지만, 간호사가 경험하는 투약근접오류 감소 효과를 확인한 경우는 거의 없었다. 이에 본 연구에서 의사결정 교육으로 중환자실 간호사의 투약근접오류 감소 효과를 검증한 점은 의미가 있다.

투약오류 예방을 위해서는 투약지식 습득은 물론이고, 습득한 지식을 적용한 수행 연습과 성찰 과정의 반복적인 순환 절차가 이뤄져야 한다[30]. 반복 수행과 성찰 경험은 환자안전 동기를 유발하여[30] 행위의 적용과 지속을 촉진하기에, 본 연구에서는 대상자에게 4개의 연속된 상황이 전개되는 시나리오를 활용한 시뮬레이션 교육을 제공하였다. 이를 통해 본 연구의 대상자가 중환자실 고위험약물 투약오류 복구 수행을 반복 경험하도록 하였고, 그 결과 중재 이후 임상 현장에서 올바른 임상의사결정을 통한 고위험약물 투약오류 복구를 실천할 것을 기대하였다. 임상의사결정 프로그램 적용 3개월 후 대상자의 투약근접오류 경험 감소는 대상자가 임상에서 올바른 의사결정을 적용한 결과로 사료되며, 이는 중환자실 고위험약물 투약안전 측면에서 의미가 있다.

의사결정 훈련 2주 후 간호사의 임상의사결정능력과 투약안전역량 향상 효과를 확인한 선행연구에서[10]는 중재 2주 이후의 지속 효과는 검증하지 않았다. 간호사의 지식과 태도 변화보다 문제해결능력과 임상의사결정능력 향상에 더 많은 시간이 소요됨을 고려할 때[12], 간호사 대상 고위험약물 투약 상황에서의 올바른 임상의사결정 훈련 효과가 지속되어 투약근접오류 감소 효과가 나타나기까지 2주보다 더 많은 시간이 소요될 것으로 예상되었다. 이에 본 연구는 간호사의 투약오류 경험을 조사한 선행연구[16]를 근거로 중재 3개월 후 투약근접오류 경험을 조사하였고 중재의 지속 효과를 확인하였다. 이는 간호사의 임상의사결정 훈련이 중재 3개월까지는 투약근접오류 감소에 효과를 미치고 지속됨을 의미하는 것으로, 본 연구결과를 통해 간호사 대상 투약근접오류 감소 예방 중재의 재교육 시기 선정 근거를 설정할 수 있겠다.

본 연구결과 중환자실 간호사의 고위험약물 투약오류 복구수준은 높지 않았다. 고위험약물 투약오류 예방 임상의사결정 프로그램에 참여한 실험군의 시나리오 수행 전체 평균 점수는 100점 만점에 51.92점, 최하 점수 34.72점, 최고 점수 65.28점이었다. 전체 시나리오를 경험하는 동안 고위험약물 투약오류 복구를 모두 수행하여 투약오류 발생 없이 투약 수행을 마친 경우는 하나도 없었고, 모든 시나리오에서 고위험약물 투약오류가 발생하였다. 같은 시나리오를 적용한 선행연구가 없어 비교는 어려우나, 이는 투약 시뮬레이션 교육으로 고위험약물 투여지침 위반 처방에 대한 간호사 오류복구를 조사한 연구에서 고위험약물 구두 처방에 문제를 제기한 대상자가 하나도 없었던 선행연구 결과[14]와 맥락을 같이 한다. 선행연구의 연구대상이 입사 3년 이내 일반 병동간호사임을 고려할 때[14] 고위험약물을 빈번하게 사용하는 중환자실 전체 간호사를 대상으로 한 본 연구 대상자의 고위험약물 투약오류 복구수준은 환자안전 측면에서 높다고 할 수 없다. 이는 고위험약물 투약지식과 자신감이 간호사의 임상경력과 투약 경험이 증가됨에 따라 저절로 획득되지 않는다는 선행연구 결과[11]를 뒷받침하는 것으로, 저연차 간호사를 대상으로 교육이 적용되었던 선행연구와 달리[10,11], 고위험약물 투약안전과 관련하여서는 전체 경력간호사에게 체계적으로 교육이 적용되고 관리되어야 함을 시사하고 있다.

또한, 간호사의 고위험약물 투약오류 복구수준에 대한 연구결과는 한 치의 실수나 오류가 허용되지 않는 고위험약물 투약오류가 흔히 발생할 수 있음을 의미한다[14]. 이는 단시간 시뮬레이션 훈련으로 간호사의 고위험약물 투약오류 복구수준을 평가하고 역량을 증진시키는데 한계가 있음을 확인한 결과[14]로서 간호사의 고위험약물 투약오류 복구에 대한 지속적인 모니터링과 반복 교육이 필요함을 시사한다. 또한, 본 연구에서는 대상자의 오류복구 행위를 자가보고가 아닌 행위 관찰을 통해 확인하였다. 이는 임상 투약 상황에서 투약안전에 위협이 되는 행위를 직접 관찰하였다는 점에서 의미가 있다. 이상 연구결과를 통해 고위험약물 투약안전을 위한 다양한 전략을 마련할 수 있다. 먼저 오류복구 체크리스트를 활용한 시뮬레이션 교육은 간호사에게 자신의 투약오류 발생 위험을 성찰하여 교정할 기회를 제공한다. 또한, 교수자에게는 간호사의 투약안전 위협 행위에 대한 직접 관찰로, 학습자가 투약오류 예방을 위해 수정할 점을 확인하고 지도하는데 활용될 수 있다. 즉, 간호사의 투약안전 역량 강화를 위한 교육에서 유용하게 활용될 수 있기에 본 연구의 오류복구 체크리스트 개발은 의미가 있다. 또한, 오류복구 체크리스트는 임상 실무에서 간호사의 투약 수행 관리에도 활용될 수 있다. 간호사가 처방, 조제 및 투약 전 과정에서의 투약오류 상황을 복구하여 안전한 투약 수행을 하도록 실무 투약 현장에서 체크리스트를 활용하여 주기적으로 관찰하고, 점검한다면 이들의 투약안전 수행 역량을 높이는데, 도움이 될 것이다.

본 연구는 몇 가지 제한점이 있다. 첫째, 일개 지역 일 상급종합병원 간호사를 대상으로 연구가 수행되었기 때문에 일반화에 주의가 필요하다. 둘째, 실험설계에서 실험자 효과를 줄여 내적타당도를 높이기 위해 자료수집자에게 대상자 할당군을 밝히지 않는 단일 맹검법을 적용하였으나 연구자와 중재자 맹검법을 적용하지 못하였다. 셋째, 대상자의 투약근접오류 경험 횟수를 자가보고로 측정하여 주관성을 배제하지 못하였다.

## 결론

본 연구는 고위험약물 투약오류 예방 임상의사결정 프로그램을 개발하고, 효과를 확인하였다. 연구결과 임상의사결정 프로그램은 중환자실 간호사의 임상의사결정능력 향상, 고위험약물 투약오류 위험수준과 투약근접오류 경험 감소에 효과가 있었다. 따라서, 임상의사결정 프로그램의 적용으로 중환자실 고위험약물 투약상황에서 간호사의 올바른 임상의사결정을 통한 고위험약물 투약오류 예방과 안전한 투약 수행을 기대할 수 있다는 점에서 의의가 있다.

이상의 결과를 근거로 추후 연구 방향을 위해 다음을 제언하고자 한다. 첫째, 투약근접오류 경험을 자가 보고로 측정하여 주관성을 배제하지 못하였으므로 객관적 측정 지표를 활용한 연구 시도를 제언한다. 둘째, 임상의사결정 교육의 교수학습방법으로 사례기반학습 단일 중재의 효과를 검증하기 위한 연구 시도를 제언한다. 셋째, 임상의사결정 프로그램 적용 대상자의 고위험약물 투약오류 및 오류복구 경험을 탐색하고 중재 효과를 보완, 설명하기 위하여 학습자 경험에 대한 질적 데이터 수집을 추가하는 혼합설계로의 연구 진행을 제언한다.

## Figures and Tables

**Table 1.** Inter-rater Reliability of a Checklist for Recovering High-alert Medication Errors in Intensive Care Units and Scores for Recovering High-alert Medication Errors in Each Scenario

Scenario	ICC(2,1)	95% CI	*p*	Scores for recovering high-alert medication errors			
				M ± SD	Min	Max	Range
1	.97	.90~.99	< .001	45.00 ± 0.90	35.42	56.25	0.00~100.00
2	.85	.43~.96	.005	55.91 ± 0.94	45.45	63.64	0.00~100.00
3	.96	.83~.99	< .001	56.50 ± 0.80	52.50	62.50	0.00~100.00
4	.90	.74~.97	< .001	50.28 ± 0.82	34.72	65.28	0.00~100.00

ICC = Intraclass correlation coefficients; CI = Confidence interval; M = Mean; SD = Standard deviation; Min = Minimum; Max = Maximum.

**Table 2.** Development and Implementation Based on the ADDIE Model

ADDIE model	Program development and implementation in this study			
Analysis	Literature review (total of 15 studies)			
	Learner and environment analysis			
↓	↓			
Design	Setting goals based on literature review			
	① To reduce the risk level of high-alert medication errors			
	② To improve clinical decision-making ability			
	③ To reduce the experience of near-miss medication errors			
	Selection of evaluation tools			
	Intervention strategies			
	① Program content composition: Simon’s decision-making model			
	② Program teaching method : High-fidelity simulation			
↓	↓			
Development	• Development of the program			
	• Expert content validity and learner validity			
	• Modification of program			
	• Final program (total 4 scenarios for 180 minutes)			
	Scenario	Briefing	Running a simulation scenario	Debriefing based on GAS model
	1	10 minutes	10 minutes	20 minutes
	2	6 minutes	10 minutes	20 minutes
	3	7 minutes	15 minutes	30 minutes
	4	7 minutes	15 minutes	30 minutes
↓	↓			
Implementation	• Control group: Lecture (total 90 minutes)			
	• Experimental group: Intervention (total 180 minutes)			
↓	↓			
Evaluation	• Pre-test (at least 2 weeks prior to intervention or lecture)			
	• Post-test 1 (immediately after intervention)			
	• Post-test 2 (3 months after intervention)			

ADDIE = Analysis, design, development, implementation, evaluation; GAS = Gather, analyze, summarize.

**Table 3.** Simulation-based Clinical Decision-making Program for Preventing High-alert Medication Errors

No.	Scenario topic (complexity)	Examples of error situation within a scenario	Examples of error recovery steps in each simulation scenario	Clinical decision-making processes (intelligence-design-choice) according to error recovery steps
1	Medication errors due to KCL (simple)	KCL was prescribed for peripheral vein infusion, but the prescribed concentration exceeded the maximum dilution.	Recognize misprescribing by reviewing high-alert medication prescribing guidelines and KCL pharmacy information	Intelligence applied to error recognition and interruption
			Plan error recovery by requesting correction of incorrect prescriptions based on evidence	Design applied to error recovery plan
			Re-examine the accuracy of the revised prescription	Choice applied to implementing and evaluating error recovery
			Perform safe medication according to the prescription	
2	Medication errors due to insulin (simple)	Wrong insulin (Insulin lispro→Regular insulin) dispensed by the pharmacy.	Recognize discrepancies between the medication card and the medication by checking medication information and appearance-related medication guidelines	Intelligence applied to error recognition and interruption
			Plan to check the pharmacy and drug delivery process to ensure that any drugs that are incorrectly dispensed due to similar appearance are replaced with the correct medication	Design applied to error recovery plan
			Reconfirm that the replacement drug is the correct medication	Choice applied to implementing and evaluating error recovery
			Perform safe medication according to the prescription	
3	Medication errors due to KCL & insulin when nursing needs occur simultaneously (complex)	While patient A is receiving continuous insulin infusion, patient B is prescribed rapid fluid infusion (KCL mixed fluid) due to hypotension	Recognize that in situations where nursing needs arise for another patient B it may be necessary to temporarily stop the insulin infusion for patient A or to ask for assistance	Intelligence applied to error recognition and interruption
			Recognized KCL infusion not suitable for rapid administration in patient B by reviewing high-alert medication administration guidelines	
			Plan to temporarily stop patient A's insulin infusion or ask for help in a situation where another patient B needs nursing	Design applied to error recovery plan
			Plan to request a change in prescription because prescribing rapid infusion of KCL mixed fluid for patient B is not appropriate	
			Re-examine the accuracy of the revised prescription for patient B & perform safe medication according to the prescription	Choice applied to implementing and evaluating error recovery
			Resume safe insulin infusion for patient A with a glucose check	
4	Medication errors due to moderate sedation & neuromuscular blockade in emergency situations (complex)	In the event of hemodynamic instability, moderate sedatives and neuromuscular blockers are prescribed	Recognize the risk of administering sedatives to hemodynamically unstable patients by checking the high-alert medication administration guidelines	Intelligence applied to error recognition and interruption
			Planning to administer medication after securing the patient's hemodynamic stability before administering high-alert medication	Design applied to error recovery plan
			Proceed with medication after confirming the patient's hemodynamic stability	Choice applied to implementing and evaluating error recovery
			Perform safe medication according to the prescription	

KCL = Potassium chloride.

**Table 4.** Homogeneity of General Characteristics and Dependent Variables Between Groups (N = 40)

Characteristics	Categories	Exp (n = 20)	Con (n = 20)	*χ*^2^/t/Z	*p*
		n (%) or M ± SD			
General characteristics					
Sex	Men	7 (17.5)	5 (12.5)	0.48^†^	.731
	Women	13 (32.5)	15 (37.5)		
Age (year)		28.40 ± 3.76	30.50 ± 7.38	－0.61^‡^	.541
Education	Bachelor’s degree	19 (47.5)	17 (42.5)	1.11^†^	.605
	≥ Master’s degree	1 (2.5)	3 (7.5)		
Total clinical career (months)		54.35 ± 45.95	78.05 ± 82.46	－1.16^‡^	.244
Career in current department (months)		34.15 ± 22.66	42.90 ± 31.16	－0.83^‡^	.414
Dependent variables					
Clinical decision-making ability		139.90 ± 7.41	136.80 ± 9.45	1.16	.255
Risk level of high-alert medication errors		1.58 ± 0.22	1.59 ± 0.19	－0.49^‡^	.622
Experience of near-miss medication errors		9.80 ± 13.79	3.50 ± 5.69	－1.09^‡^	.275

Exp = Experimental group; Con = Control group; M = Mean; SD = Standard deviation.

† Fisher’s exact test;

‡ Mann-Whitney U test.

**Table 5.** Effects of the Intervention on Clinical Decision-making Ability, Risk of High-alert Medication Errors, and Experience of Near-miss Medication Errors (N = 40)

Variables	Group	Pre-test	Post-test 1	Post-test 2	Sources	χ^2^	*p*
		M ± SD					
Clinical decision-making ability	Exp (n = 20)	139.90 ± 7.41	151.80 ± 13.77	145.20 ± 8.60	G	9.30	.002
					T	27.37	< .001
	Con (n = 20)	136.80 ± 9.45	139.50 ± 8.70	138.45 ± 7.48	G×T	10.57	.005
Risk level of high-alert medication errors	Exp (n = 20)	1.58 ± 0.22	1.39 ± 0.17	1.31 ± 0.16	G	4.08	.043
					T	33.72	< .001
	Con (n = 20)	1.59 ± 0.19	1.51 ± 0.21	1.48 ± 0.26	G×T	6.17	.046
Experience of near-miss medication errors	Exp (n = 20)	9.80 ± 13.79	N/A	2.45 ± 3.07	G	1.09	.297
					T	3.08	.079
	Con (n = 20)	3.50 ± 5.69		5.00 ± 4.55	G×T	7.05	.008

Exp = Experimental group; Con = Control group; M = Mean; SD = Standard deviation; G = Group; T = Time; N/A = Not available.

## Data Availability

Please contact the corresponding author for data availability.
